# Immunomodulatory Activity In Vitro and In Vivo of a Sulfated Polysaccharide with Novel Structure from the Green Alga *Ulva* *conglobata* Kjellman

**DOI:** 10.3390/md20070447

**Published:** 2022-07-08

**Authors:** Sujian Cao, Yajing Yang, Shan Liu, Zhuling Shao, Xiao Chu, Wenjun Mao

**Affiliations:** 1 Advanced Medical Research Institute, Shandong University, Jinan 250012, China; 201990000083@sdu.edu.cn; 2 Key Laboratory of Marine Drugs of Ministry of Education, Shandong Key Laboratory of Glycoscience and Glycotechnology, School of Medicine and Pharmacy, Ocean University of China, Qingdao 266003, China; yangyajing@stu.ouc.edu.cn (Y.Y.); 11210811010@stu.ouc.edu.cn (S.L.); shaozhuling@stu.ouc.edu.cn (Z.S.); 21200831151@stu.ouc.edu.cn (X.C.)

**Keywords:** alga, sulfated polysaccharide, structural characteristics, immunomodulatory activity

## Abstract

Algae accumulate large amounts of polysaccharides in their cell walls or intercellular regions. Polysaccharides from algae possess high potential as promising candidates for marine drug development. In this study, a sulfated polysaccharide, UCP, from the green alga *Ulva conglobata* Kjellman was obtained by water extraction, anion-exchange, and size-exclusion chromatography purification, and its structure was characterized by a combination of chemical and spectroscopic methods. UCP mainly consisted of →4)-α/β-l-Rha*p*-(1→, →4)-β-d-Xyl*p*-(1→ and →4)-β-d-GlcA*p*-(1→ residues. Sulfate ester groups were substituted mainly at C-3 of →4)-l-Rha*p*-(1→ and C-2 of →4)-β-d-Xyl*p*-(1→. Partial glycosylation was at C-2 of →4)-α-l-Rha*p*-(1→ residues. UCP possessed a potent immunomodulatory effect in vitro, evaluated by the assays of lymphocyte proliferation and macrophage phagocytosis. The immunomodulatory activity of UCP in vivo was further investigated using immunosuppressive mice induced by cyclophosphamide. The results showed that UCP markedly increased the spleen and thymus indexes and ameliorated the cyclophosphamide-induced damage to the spleen and thymus. UCP could increase the levels of white blood cells, lymphocytes, and platelets, and improve the hematopoietic inhibition caused by cyclophosphamide. Moreover, UCP significantly promoted the secretions of the immunoglobulin (Ig)G, IgE, and IgM. The data demonstrated that UCP is a novel sulfated polysaccharide and may be a promising immunomodulatory agent.

## 1. Introduction

Algae are an efficient producer of polysaccharides. Algal polysaccharides have attracted increasing attention due to their various biological functions, such as anticoagulant, antitumor, immunomodulatory activity, antiviral, and antioxidant [1,2,3,4,5,6]. So far, extensive studies on polysaccharides from brown and red algae such as fucoidans, agar, and carrageenan have been carried out. However, the investigations on polysaccharides from green algae are significantly fewer than those from brown and red algae [7].

In recent years, polysaccharides with unique structures from green algae have been reported to have various biological activities, including immunomodulatory activity [8,9]. Polysaccharides from *Chlorella* sp. exhibited strong immunomodulatory activities by inducing the release of interleukin-1, nitric oxide, and prostaglandin-2 [10,11,12,13]. Lee et al. [14] found that the polysaccharide from *Codiun fragile* possessed potent immunostimulatory activity by activating macrophages while preventing potential detrimental inflammatory effects from excessive macrophage activation. Hao et al. [15] reported that the polysaccharide CRVP-1 from *Caulerpa racemosa var peltate* had obvious effects on the macrophage proliferation and production of cytokines. Polysaccharides from *Enteromorpha intestinalis* could stimulate concanavalin A (ConA)-induced lymphocyte proliferation and activate macrophages to release tumor necrosis factor α [16]. In addition, an increasing number of reports revealed that the polysaccharides from green algae, *Ulva* species, possessed immunomodulatory activity [17,18,19,20,21]. Polysaccharides from green algae belong to a group of what may be the most promising immunomodulatory compounds.

*Ulva conglobata* Kjellman is an edible green alga that is widely distributed through the world’s seas. In this study, a novel sulfated polysaccharide was obtained from *U. conglobata* Kjellm, and its structure was characterized by a combination of chemical and spectroscopic methods. The immunomodulatory activity in vitro and in vivo of the sulfated polysaccharide was investigated. The sulfated polysaccharide could have high potential as immunomodulatory agent.

## 2. Results and Discussion

### 2.1. Chemical Composition of UCP

**Figure 1:**
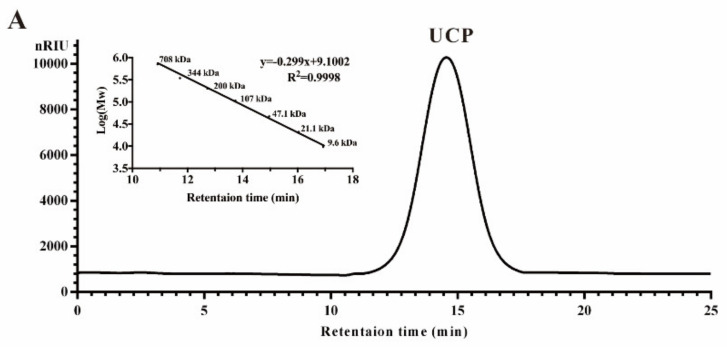

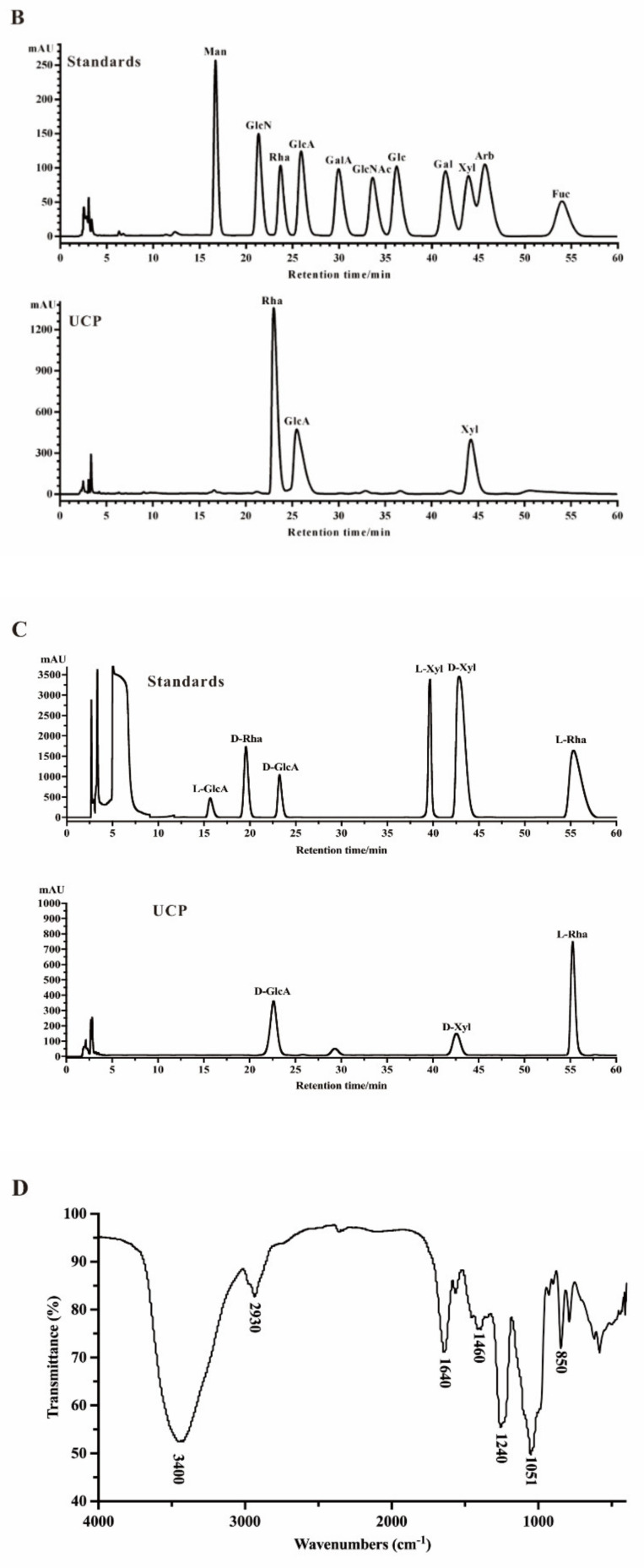
HPGPC chromatogram, HPLC chromatogram, and IR spectrum of UCP. (**A**) HPGPC chromatogram of UCP on a Shodex OHpak SB-804 HQ column, and the standard curve of molecular weight; (**B**) HPLC chromatogram for monosaccharide composition analysis of UCP (Man: d-mannose, GlcN: d-glucosamine, Rha: l-rhamnose, GlcA: d-glucuronic acid, GalA: d-galacturonic acid, Glc: d-glucose, Gal: d-galactose, Xyl: d-xylose, Ara: l-arabinose, Fuc: l-fucose); (**C**) HPLC chromatogram of the sugar configuration determination of UCP (d-Rha: D-rhamnose, d-GlcA: d-glucuronic acid, d-Xyl: d-xylose, l-Rha: l-rhamnose, l-GlcA: l-glucuronic acid, l-Xyl: l-xylose); (**D**) IR spectrum of UCP.

Crude polysaccharide was extracted from *U. conglobata* Kjellman by hot water and then fractionated using a Q Sepharose Fast Flow column. The fraction eluted with 0.5 mol/L NaCl was further purified by a Sephacryl S-400/HR column. Finally, a polysaccharide fraction, designed UCP, was obtained. The yield of UCP from starting material was about 1.45% (*w*/*w*). UCP appeared as a single and symmetrical peak in a high performance gel permeation chromatogram (HPGPC) (Figure 1A), and its average molecular weight was about 67.80 kDa based on its retention time in the HPGPC chromatogram. The monosaccharide composition analysis (Figure 1B) showed that UCP consisted of rhamnose, glucuronic acid, and xylose, with a mole ratio of 2.79:1.19:1.00. The sugar configuration analysis showed that rhamnose was in the l-configuration, whereas glucuronic acid and xylose were in the d-configuration (Figure 1C). The sulfate ester content of UCP was 30.27%, and the glucuronic acid content was 21.45%. No protein was found in UCP.

A Fourier-transform infrared (FTIR) spectrum of UCP is shown in Figure 1D. The characteristic band at 3400 cm^−^^1^ was attributed to the O–H stretching vibrations, and the absorption band at 2930 cm^−^^1^ was due to C–H stretching vibrations. The band at 1051 cm^−^^1^ was from the stretching vibrations of C–O and change angle vibrations of O–H. The band at 1640 cm^−^^1^ corresponded to asymmetric stretch vibrations of the carboxylic group of the glucuronic acid. Furthermore, the band at 1460 cm^−^^1^ could be the stretching vibration of the carbonyl group. The relatively strong absorption peaks at 850 and 1240 cm^−^^1^ derived from the stretching vibrations of C–O–S of sulfate ester in the axial position and S–O stretching vibrations, respectively.

### 2.2. Structural Characteristics of UCP

#### 2.2.1. Methylation Analysis

In order to obtain the information of the linkage pattern and the sulfate position in UCP, a comparative methylation analysis between UCP and its desulfated product (DsUCP) was carried out. As shown in Table 1, UCP was mainly composed of →4)-Rha*p*-(1→, →2,4)-Rha*p*-(1→, →3,4)-Rha*p*-(1→, →2,4)-Xyl*p*-(1→, and →4)-Xyl*p*-(1→ residues, with minor amounts of T-Rha*p* and T-Xyl*p* residues. Compared with the methylation results of UCP, the amounts of →4)-Rha*p*-(1→ and →4)-Xyl*p*-(1→residues were increased, whereas →3,4)-Rha*p*-(1→ and →2,4)-Xyl*p*-(1→ residues disappeared in DsUCP. Moreover, no →3)-Rha*p*-(1→ and →2)-Xyl*p*-(1→residues were detected. Thus, the sulfate ester groups were at the C-3 of →4)-Rha*p*-(1→ and C-2 of →4)-Xyl*p*-(1→ residues. The presence of →2,4)-Rha*p*-(1→ in DsUCP showed that UCP had side chains at C-2 of the →4)-Rha*p*-(1→ residue. In order to obtain the information about the linkage pattern of glucuronic acid, methylation analysis was also carried out with the carboxyl-reduced product of UCP (RdUCP). Compared with the methylation results of UCP, increased amounts of glucose in the form of →4)-Glc*p*-(1→ and T-Glc*p* were detected in RdUCP, indicating the glucuronic acid in UCP mainly existed in the form of →4)-GlcA*p*-(1→ with a minor amount of T-GlcA*p*.

#### 2.2.2. NMR Analysis of UCP

The structure of UCP was further characterized by NMR analysis. In the ^1^H NMR spectrum of UCP (Figure S1A), there were seven anomeric proton signals occurring at 5.31 (A), 5.12 (B), 5.02 (C), 4.95 (D), 4.91 (E), 4.68 (F), and 4.55 (G) ppm, with relative integrals of 1.00: 2.05:1.65: 2.74: 2.07: 3.46: 1.27. The signal at 1.35 ppm was assigned to the proton of the CH_3_ group of the rhamnose residue. Other proton signals at 3.30–4.60 ppm were H-2–H-5 of the ring protons.

In the ^13^C NMR spectrum (Figure S1B), the anomeric carbon signals occurred at 98.93–105.18 ppm. The signal at 18.42 ppm was assigned to C-6 of the rhamnose residues. The α-anomeric configuration of rhamnose residues was deduced by the H-5 signal at 4.0–4.2 ppm and the C-5 signal at 69–70 ppm. The β-anomeric configuration of rhamnose residues was also found due to the H-5 signal at 3.6–3.7 ppm and the C-5 signal at 71–72 ppm [22]. The signals at 62.75 and 63.92 ppm were attributed to C-5 of the β-anomeric configuration of xylose residues compared with that of the α-anomer at 58–61 ppm [23]. In addition, the signal at 176.46 ppm was assigned to C-6 of the carboxyl group of glucuronic acid [24]. Other signals at 70–80 ppm were attributed to C-2–C-5 of the rhamnose, xylose, and glucuronic acid residues.

The ^1^H NMR spin systems of the polysaccharide were assigned by the ^1^H–^1^H COSY spectrum (Figure S1C). The direct C–H coupling was determined by the ^1^H–^13^C HSQC spectrum (Figure S1D). Combined with the analysis of the ^1^H–^13^C HSQC spectrum and the comparison with the chemical shift data of similarly substituted sugar residues, the assignment of the main signals of the seven sugar residues could be completed. The anomeric proton signal of A at 5.31 ppm was correlated to the anomeric carbon signal at 102.59 ppm. A was assigned to →2,4)-α-l-Rha*p*-(1→ because of the downfield chemical shifts of C-2 at 79.80 and C-4 at 79.59 ppm compared with that of parent α-l-rhamnopyranose residues [25]. The proton signal at δ 5.12 ppm of the residue B was correlated to the anomeric carbon signal at 103.33 ppm. The correlated signals H3/C3 (4.67/79.80) and H4/C4 (3.84/79.49) indicated that the residue B was →4)-α-l-Rha*p*(3SO_4_)-(1→, and the α-configuration was deduced by the correlated signal of H5/C5 at 4.05/69.61 ppm. Similarly, the residue D was attributed to →4)-β-l-Rha*p*-(3SO_4_)-(1→ because of the downfield chemical shifts of H3/C3 (4.62/79.80) and H4/C4 (3.80/79.49), and the β-configuration was deduced by its H5/C5 at 3.63/71.62 ppm. The C-5 of the residue C at 62.75 ppm indicated that the residue C was β-xylose. The downfield chemical shifts of the C-2 (79.49 ppm) and C-4 (75.34 ppm) illustrated that the residue C was →4)-β-d-Xyl*p-*(2SO_4_)-(1→ as compared with the standard values for xylose [26]. The signal of E at 4.91 ppm was correlated to the anomeric carbon signal at 98.93 ppm, and E was assigned to →4)-β-l-Rha*p*-(1→ because of the downfield chemical shift of the C-4 at 79.49 ppm. The anomeric proton signal of F at 4.68 ppm was correlated to the anomeric carbon signal at 104.67 ppm, and F was attributed to →4)-β-d-GlcA*p*-(1→ due to the shifted signals of C-4 at 80.29 ppm and C-6 at 176.46 ppm. The anomeric proton signal of G at 4.55 ppm was correlated to the anomeric carbon signal at 105.18 ppm, and G was attributed to →4)-β-d-Xyl*p*-(1→ in view of the signal of C-4 shifted to 77.56 ppm. The ^1^H and ^13^C chemical shifts of UCP are listed in Table 2.

**Figure 2:**
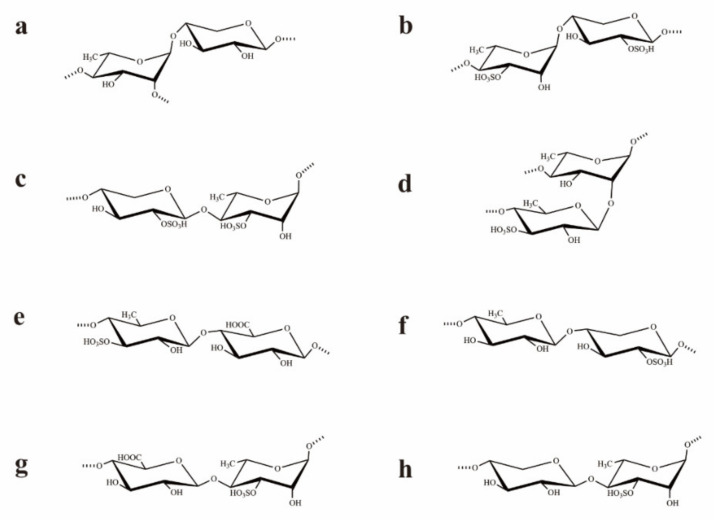
Structures of the possible main repeating disaccharides in UCP. (**a**): →2,4)-α-l-Rha*p*-(1→4)-β-d-Xyl*p*-(1→, (**b**): →4)-α-l-Rha*p*(3SO_4_)-(1→4)-β-d-Xyl*p*-(2SO_4_)-(1→, (**c**): →4)-β-d-Xyl*p*-(2SO_4_)-(1→4)-α-l-Rha*p*(3SO_4_)-(1→, (**d**): →4)-β-l-Rha*p*(3SO_4_)-(1→2,4)-α-l-Rha*p*-(1→, (**e**): →4)-β-l-Rha*p*(3SO_4_)-(1→4)-β-d-GlcA*p*-(1→, (**f**): →4)-β-l-Rha*p*-(1→4)-β-d-Xyl*p*-(2SO_4_)-(1→, (**g**): →4)-β-d-GlcA*p*-(1→4)-α-l-Rha*p*(3SO_4_)-(1→, (**h**): →4)-β-d-Xyl*p*-(1→4)-α-l-Rha*p*(3SO_4_)-(1→. These are the possible main repeating disaccharides, and some of them may not be present.

Based on the analysis of the ^1^H–^1^H NOESY spectrum (Figure S1E), the sequences of glycosyl residues in the polysaccharide chain were established. The cross signals H-1 (A)/H-4 (C/F/G) indicated the sequences →2,4)-α-l-Rha*p*-(1→4)-β-d-Xyl*p*-(2SO4)-(1→, →2,4)-α-l-Rha*p*-(1→4)-β-d-GlcA*p*-(1→, and/or →2,4)-α-l-Rha*p*-(1→4)-β-d-Xyl*p*-(1→. In fact, the cross-signal shows that at least one of the disaccharide sequences occurred, but not necessarily all three. The related signals H-1 (B)/H-4 (A/C/D/E/F/G) showed the linkages →4)-α-l-Rha*p*(3SO_4_)-(1→2,4)-α-l-Rha*p*-(1→, →4)-α-l-Rha*p*(3SO_4_)-(1→4)-β-d-Xyl*p-*(2SO_4_)-(1→, →4)-α-l-Rha*p*(3SO_4_)-(1→4)-β-l-Rha*p*(3SO_4_)-(1→, →4)-α-l-Rha*p*(3SO_4_)-(1→4)-α-l-Rha*p*-(1→, →4)-α-l-Rha*p*(3SO_4_)-(1→4)-β-d-GlcA*p*-(1→, and/or →4)-α-l-Rha*p*(3SO_4_)-(1→4)-β-d-Xyl*p*-(1→. These sequences were all consistent with the NMR data but may not have all been present. The corresponded signals H-1 (C)/H-4 (A/B/D/E) indicated the fragments as →4)-β-d-Xyl*p*(2SO_4_)-(1→2,4)-α-l-Rha*p*-(1→, →4)-β-d-Xyl*p*(2SO_4_)-(1→4)-α/β-l-Rha*p*(3SO_4_)-(1→, and/or →4)-β-d-Xyl*p*(2SO_4_)-(1→4)-β-l-Rha*p-*(1→. The disaccharide sequences may not have all been present, though their sequences were all consistent with the NMR data. The cross signals H-1 (D)/H-2 (A) and H-1 (D)/H-4(A/B/C/F/G) illustrated the sequences →4)-β-l-Rha*p*(3SO_4_)-(1→2,4)-α-l-Rha*p*-(1→, →4)-β-l-Rha*p*(3SO_4_)-(1→4)-α-l-Rha*p*(3SO_4_)-(1→, →4)-β-l-Rha*p*(3SO_4_)-(1→4)-β-d-Xyl*p*(2SO_4_)-(1→, →4)-β-l-Rha*p*(3SO_4_)-(1→4)-β-d-GlcA*p*-(1→, and/or →4)-β-l-Rha*p*(3SO_4_)-(1→4)-β-d-Xyl*p*-(1→. Similarly, at least one of the disaccharide sequences was present, but not necessarily all five. The related signals H-1 (E)/H-4 (C/E/F) indicated the sequences →4)-β-l-Rha*p*-(1→4)-β-d-Xyl*p*(2SO_4_)-(1→, →4)-β-l-Rha*p*-(1→4)-β-d-GlcA*p*-(1→, and/or →4)-β-l-Rha*p*-(1→4)-β-d-Xyl*p*-(1→. However, some of the three sequences may not have been present, though their sequences were all in agreement with the NMR data. The related signals H-1 (F)/H-2(A), H-1 (F)/H-4 (A/B/D/E) indicated the fragments →4)-β-d-GlcA*p*-(1→2,4)-α-l-Rha*p*-(1→, →4)-β-d-GlcA*p*-(1→4)-α/β-l-Rha*p*(3SO_4_)-(1→, and/or →4)-β-d-GlcA*p*-(1→4)-β-l-Rha*p*-(1→. The related signal showed that at least one of the disaccharide sequences occurred, but not all four. The corresponded signals H-1 (G)/H-2 (A) and H-1 (G)/H-4 (A/B) illustrated the linkages →4)-β-d-Xyl*p*-(1→2,4)-α-l-Rha*p*-(1→ and/or →4)-β-d-Xyl*p*-(1→4)-α-l-Rha*p*(3SO_4_)-(1→. These results illustrated that UCP was composed of →4)-α/β-l-Rha*p*-(1→, →4)-β-d-Xyl*p*-(1→, and →4)-β-d-GlcA*p*-(1→ units, with partial sulfates at the C-3 of →4)-α-L-Rha*p*-(1→ and C-2 of →4)-β-d-Xyl*p*-(1→ units. Furthermore, the cross signals H-1 (D)/H-2 (A), H-1 (G)/H-2 (A), and H-1 (F)/H-2 (A) also indicated that the branches that contained →4)-β-l-Rha*p*(3SO_4_)-(1→, →4)-β-d-GlcA*p*-(1→, and/or →4)-β-d-Xyl*p*-(1→ units were at the O-2 of →4)-β-l-Rha*p*-(1→ units. Structures of the possible main repeating disaccharides in UCP are shown in Figure 2.

The above results demonstrated that UCP was constituted by →4)-α/β-l-Rha*p*-(1→, →4)-β-d-Xyl*p*-(1→, and →4)-β-d-GlcA*p*-(1→ residues, and sulfate groups were mainly at C-3 of →4)-α/β-l-Rha*p*(1→ and C-2 of →4)-β-d-Xyl*p*-(1→. Moreover, partial glycosylation was at C-2 of →4)-α-l-Rha*p*-(1→ units. The branches contained →4)-β-l-Rha*p*(3SO_4_)-(1→ units, and →4)-β-d-GlcA*p*-(1→ and/or →4)-β-d-Xyl*p*-(1→ units may be also present in the side chains.

Sulfated polysaccharides from *Ulva* species are generally composed of rhamnose, xylose, glucuronic acid, iduronic acid, and sulfate groups, with mainly repeating disaccharide units of α-l-Rha*p*-(1→4)-β-d-Xyl*p*, β-d-GlcA*p*-(1→4)-α-l-Rha*p*3S, and α-l-IdoA-(1→4)-α-l-Rha*p*3S [27]. Other types of glycosidic bonds are also found in *Ulva* polysaccharides, such as 1,2,3-1inked rhamnose and 1,3-1inked xylose [28]. In addition, some sulfated polysaccharides from *Ulva* species contain mannose, glucose, galactose, or arabinose [29,30]. Our results showed that UCP from *U. conglobata* Kjellman possessed different structural characteristics from the sulfated polysaccharides isolated from the genus *Ulva*, though it had the repeated disaccharide in most of the *Ulva* species. The chemical structures of sulfated polysaccharides from the genus *Ulva* have great variability and complexity. The structural characteristics of algal sulfated polysaccharides depend on various factors including species, growth environment, collection site, harvest time, and extraction methods. The structural complexity of sulfated polysaccharides from *Ulva* species could be attributed to their biosynthesis machinery. The present results revealed that *Ulva* species of green algae could be a potential source of sulfated polysaccharides with novel structures.

### 2.3. Effect of UCP on Lymphocyte Proliferation

**Figure 3:**
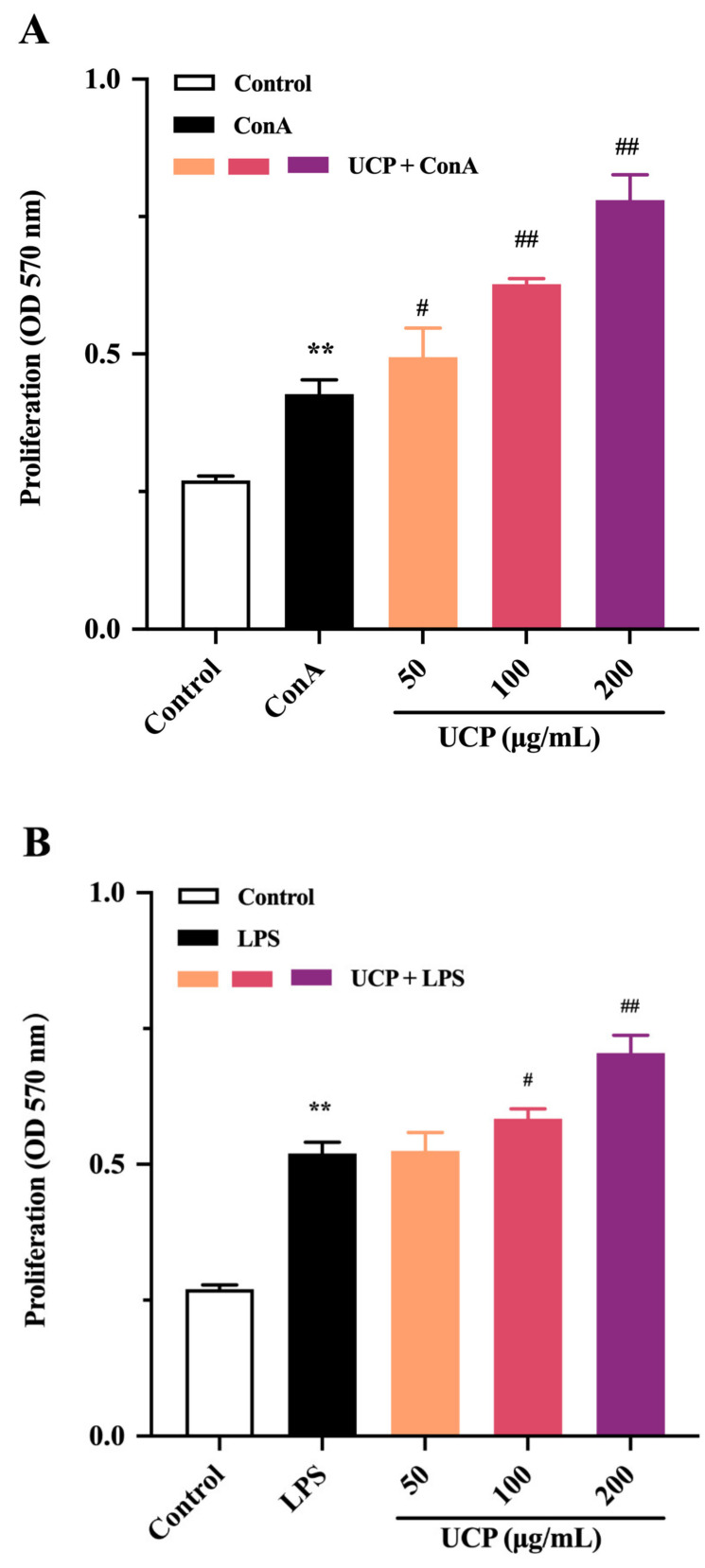

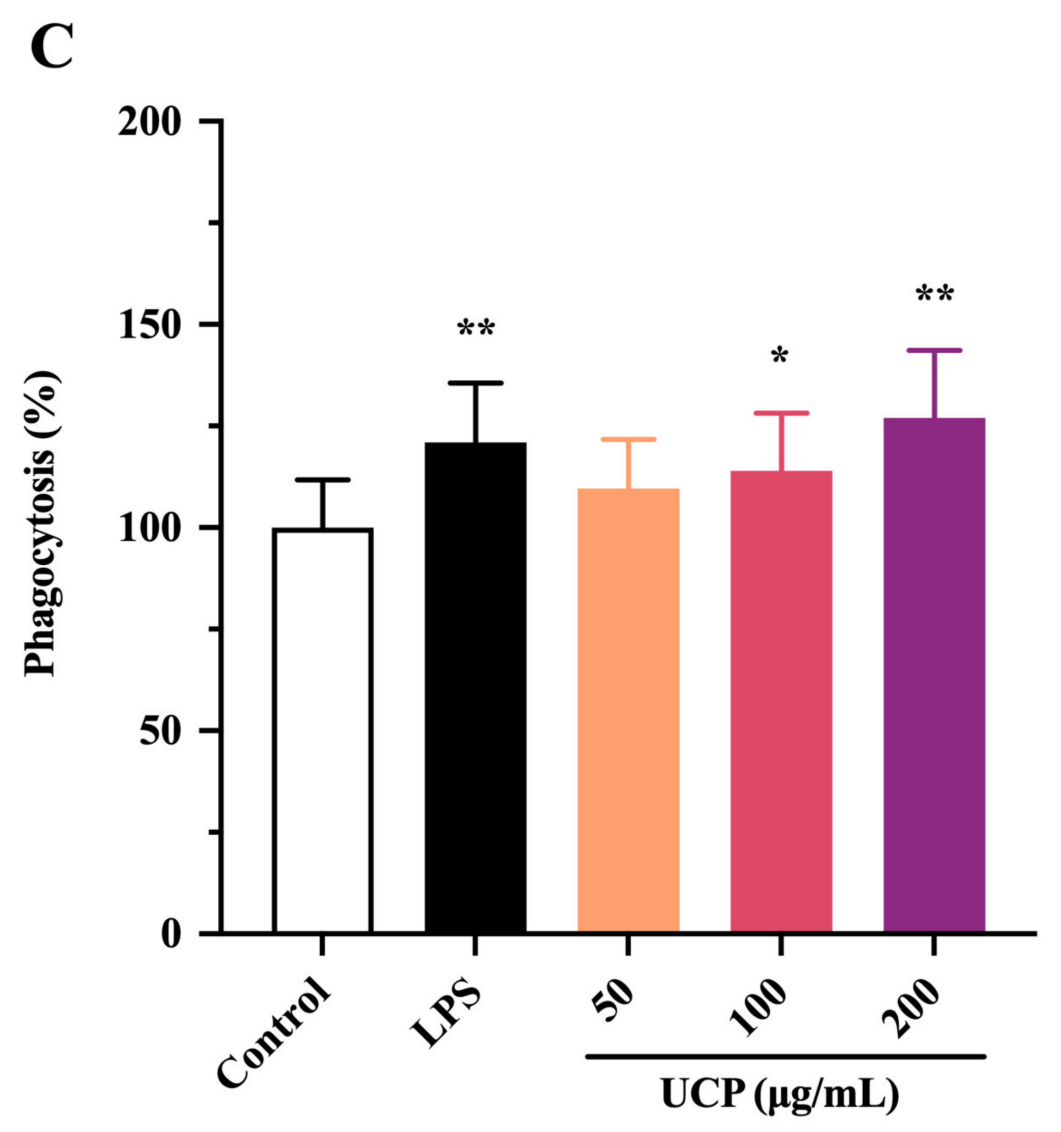
Effects of UCP on lymphocyte proliferation and macrophage phagocytosis in vitro. (**A**) Spleen cell proliferation was treated with 5 μg/mL Con A; (**B**) spleen cell proliferation was treated with 20 μg/mL LPS; and (**C**) macrophage phagocytosis. Values are mean ± standard deviation (SD) (*n* = 3). Significant: * *p* < 0.05, ** *p* < 0.01, ^#^ *p* < 0.05; ^##^ *p* < 0.01 vs. control group. OD: optical density.

The immunomodulatory activity of UCP was firstly evaluated by the proliferation ability of lymphocytes from mouse spleen. As shown in Figure 3A,B, the lymphocyte proliferation of UCP occurred in a concentration-dependent manner in the presence of Con A or lipopolysaccharide (LPS). At 200 μg/mL, UCP significantly increased the lymphocyte proliferation. Moreover, the lymphocyte proliferation of UCP was more noticeable when UCP was co-treated with ConA than with LPS at the same concentration. Lymphocyte proliferation is a pivotal event in the activation cascade of both cellular and humoral immune responses. Lymphocyte proliferation is dependent on mitogen. T cells are responsive to Con A, while LPS mainly stimulates B cell proliferation [31]. ConA can trigger the proliferation of T lymphocytes and is an important indicator of cellular immunity. LPS causes polyclonal activation of B lymphocytes, which indicates the ability of humoral immunity [32,33]. The present results showed that UCP could significantly stimulate the proliferation of T and B lymphocytes induced by ConA or LPS, illustrating that UCP could induce humoral immunity and cellular immunity to enhance the body’s immune function.

### 2.4. Effect of UCP on Phagocytic Activity of RAW246.7 Cells

The effect of UCP on the phagocytic activity of RAW264.7 (mouse monocyte macrophage leukemia cells) was also studied using a neutral red uptake assay. As shown in Figure 2C, after 24 h incubation, the phagocytic activity of RAW264.7 cells treated by UCP occurred in a dose-dependent manner. At the concentration of 50 ug/mL, the effect of UCP was not obvious compared with the control group. However, the phagocytic activity was significantly raised with increasing concentrations of UCP. At the concentration of 200 ug/mL, the phagocytosis activity of UCP exceeded that of LPS. Macrophages are the first line of defense against pathogens in the immune system, and they are involved in both specific immune and non-specific immune responses. Thus, macrophages have an important effect in the immune responses of hosts [34]. UCP could enhance the phagocytic activity of RAW264.7 cells, illustrating that UCP had the ability to activate the phagocytic activity of macrophages and increase immune responses.

Taken together, UCP could effectively increase the lymphocyte proliferation and phagocytic activity of macrophages in vitro. UCP had a positive regulatory effect on the innate immunity and nonspecific immunity. Further, the immunomodulatory effect of UCP in vivo was investigated using immunosuppressive mice induced by cyclophosphamide.

### 2.5. Immunomodulatory Activity In Vivo of UCP

Cyclophosphamide is an important chemotherapeutic drug for antitumor therapy but with serious side effects such as immunosuppression and myelosuppression by damaging DNA of normal cells [35,36,37]. In the investigation, the immunosuppressive mice induced by cyclophosphamide were used for investigating the immunomodulatory activity in vivo of UCP.

#### 2.5.1. Effect of UCP on Thymus and Spleen Indices

**Figure 4:**
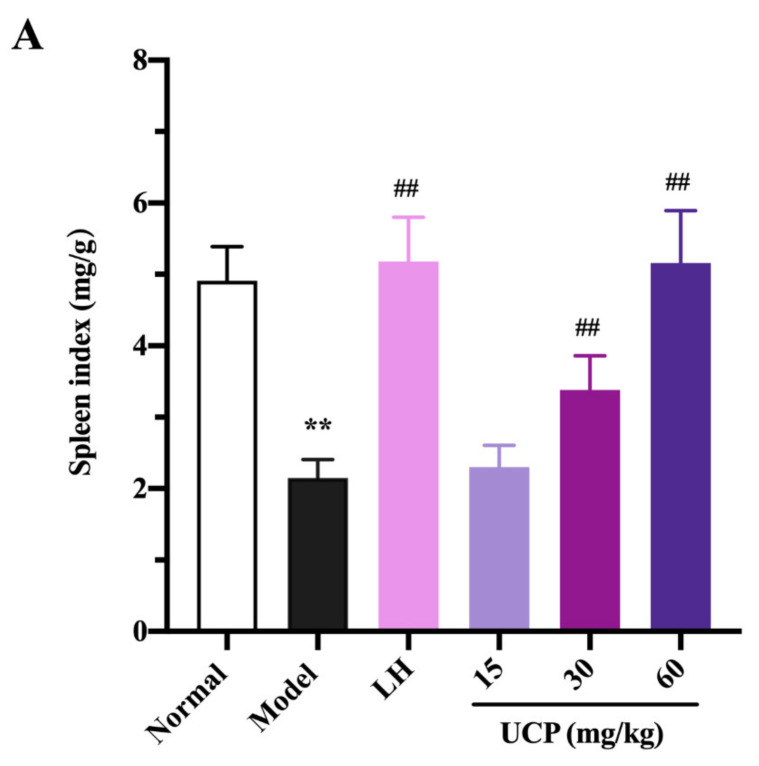

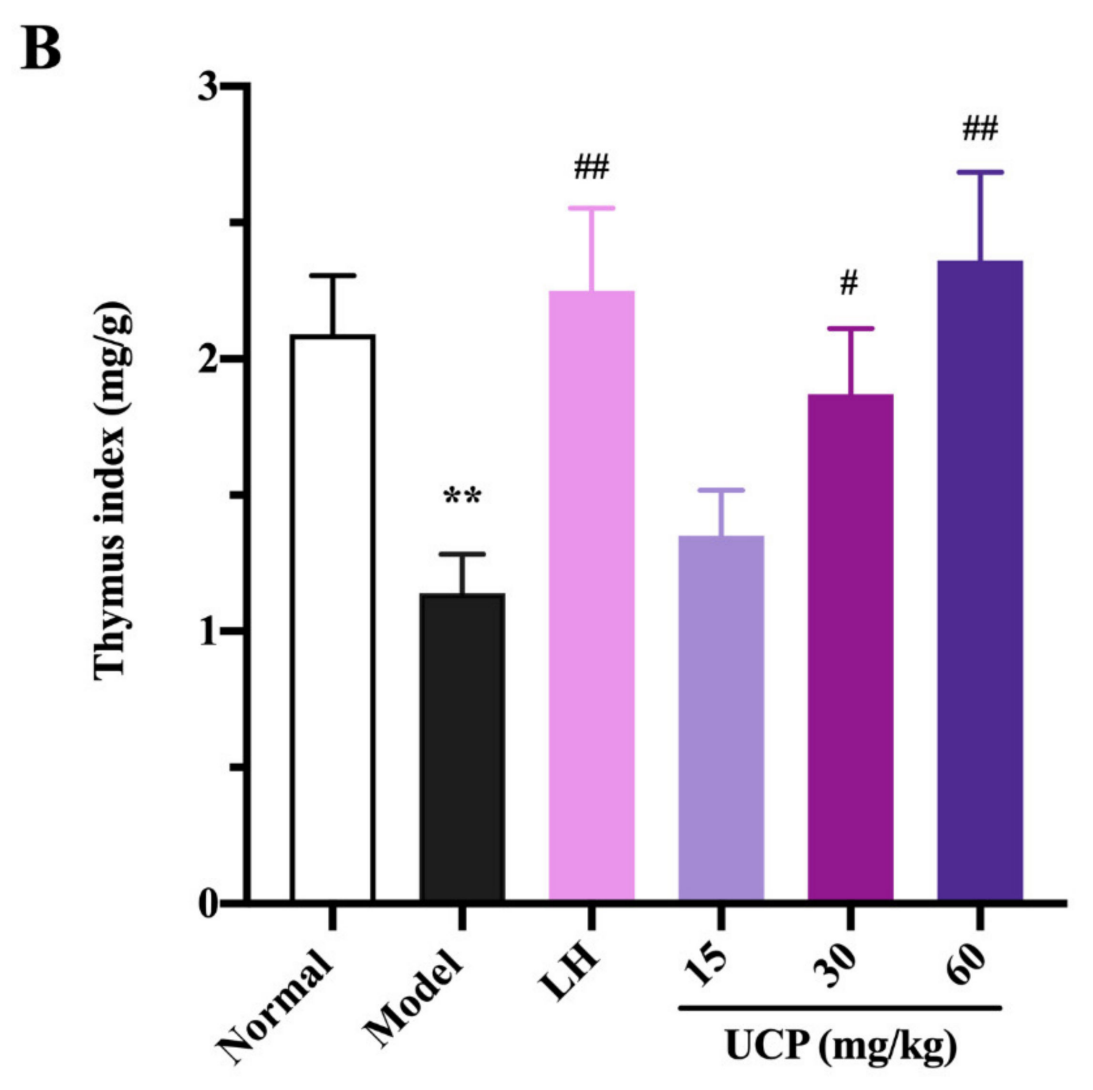
Influences of UCP on the spleen index (**A**) and thymus index (**B**) in the immunosuppressive mice induced by cyclophosphamide. The data were represented as the means ± SD (*n* = 10). Significant: ** *p* < 0.01 vs. normal group; ^#^ *p* < 0.05, ^##^ *p* < 0.01 vs. model group.

Immunomodulatory activity of UCP in vivo was firstly evaluated by thymus and spleen indices assays using levamisole hydrochloride (LH) as a reference. As listed in Figure 4, the thymus and spleen indices were significantly decreased when treated with cyclophosphamide compared with the normal group, illustrating that the immunity was disordered because of the cyclophosphamide treatment. However, it was noted that the spleen and thymus indices of UCP behaved in concentration-dependent manner. The spleen and thymus indices were effectively increased by UCP. At 60 mg/kg, the thymus and spleen indexes returned to the level of normal mice. Furthermore, it was observed that the spleen index of UCP was similar to that of LH, and the thymus index of UCP exceeded that of LH. The spleen and thymus are important immune organs and play a vital role in specific and nonspecific immunity. The spleen can eliminate older erythrocytes from circulation and cause the efficient removal of blood-borne microbes and cellular debris [38]. The thymus has a crucial influence on the establishment of adaptive immunity and a central tolerance because it mediates the maturation and choice of T lymphocytes [39]. The spleen and thymus indices can reflect the body’s immune function and immune prognosis [40]. The present result demonstrated that UCP could effectively improve the thymus and spleen function of immunosuppressive mice and exhibit strong immunomodulatory activity in vivo.

#### 2.5.2. Effect of UCP on Peripheral Blood Cells

**Figure 5:**
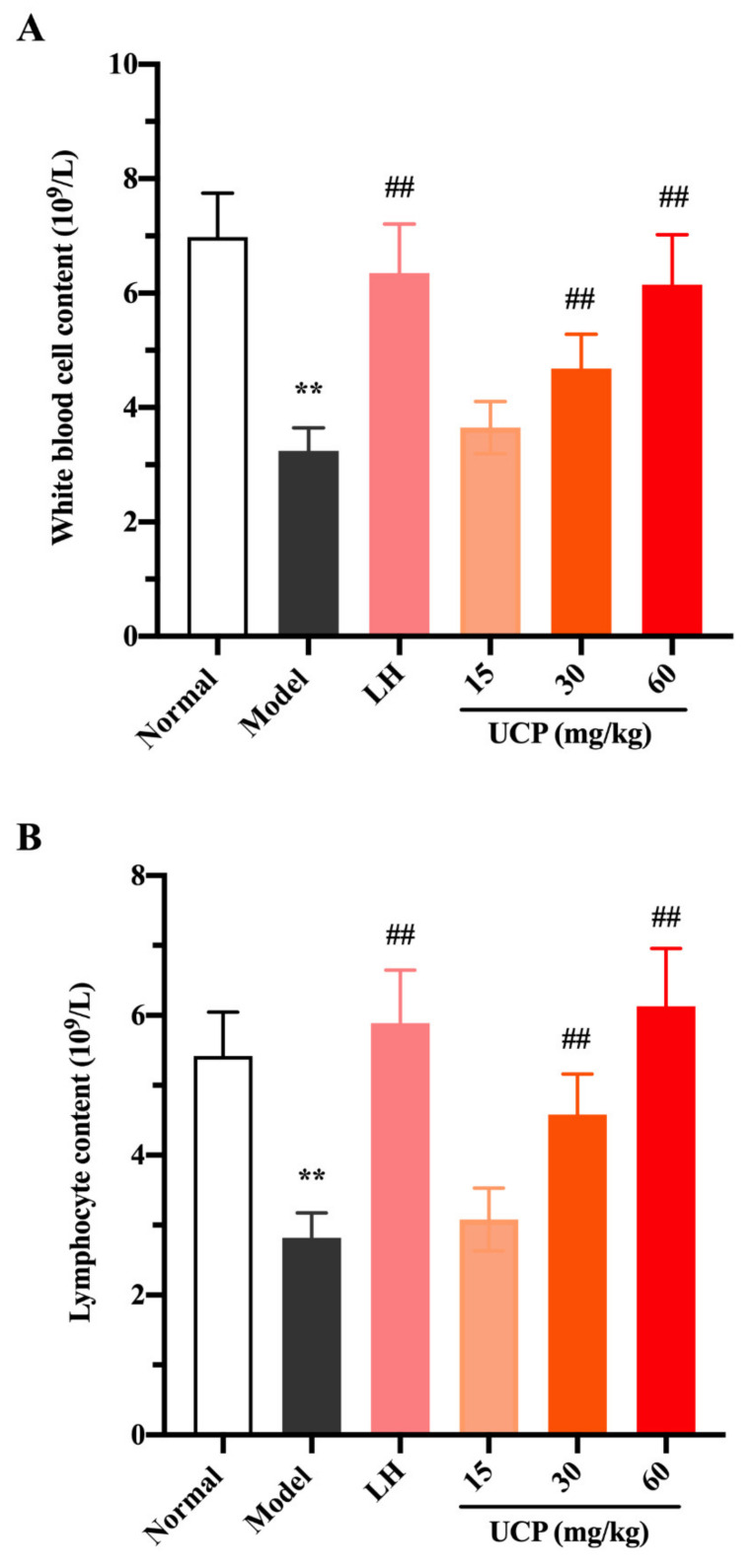

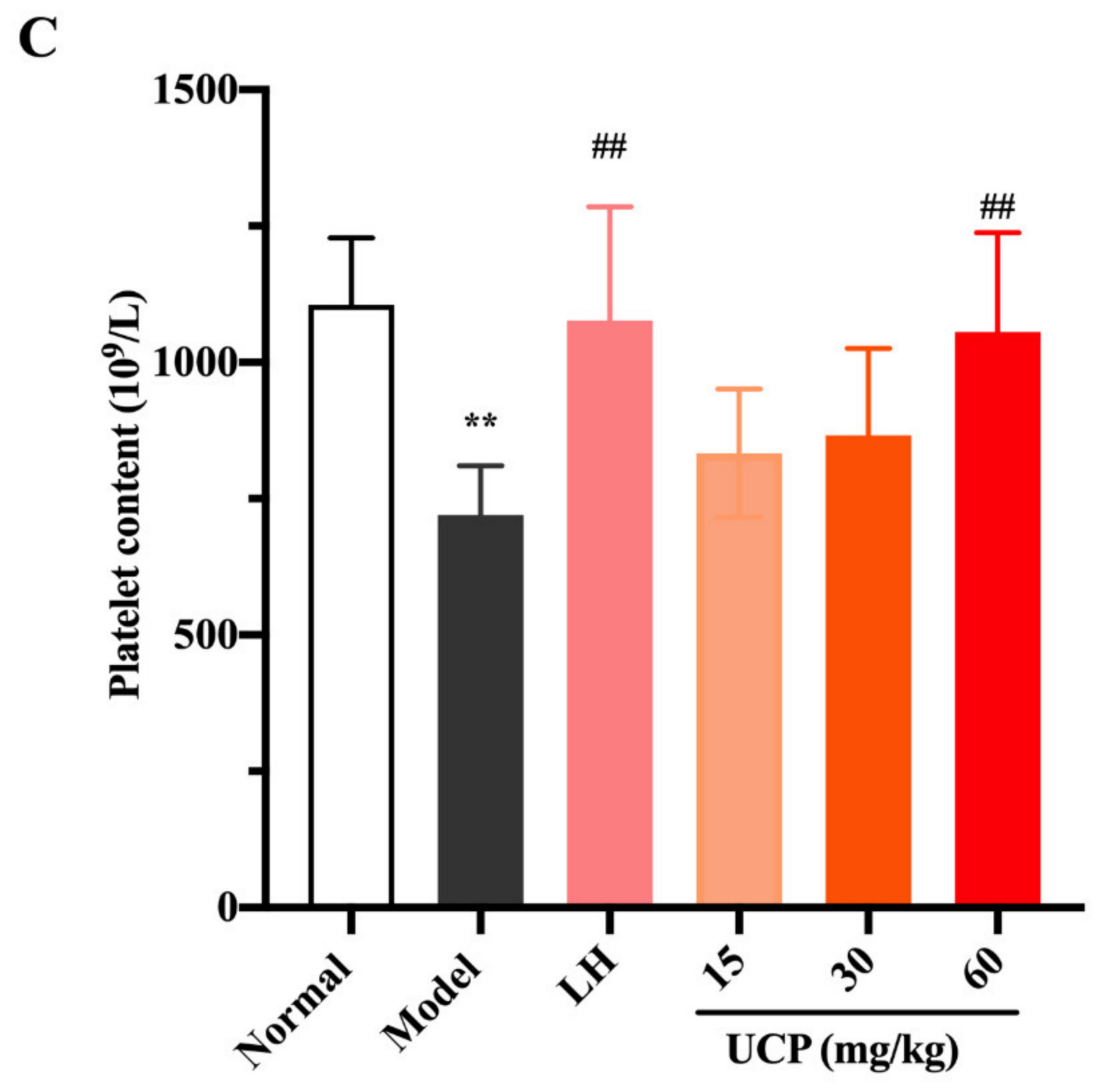
Effects of UCP on the white blood cell content (**A**), lymphocyte content (**B**), and platelet content (**C**) in immunosuppressive mice induced by cyclophosphamide. The data were represented as the means ± SD (*n* = 10). Significant: ** *p* < 0.01 vs. normal group; ^##^ *p* < 0.05 vs. model group.

White blood cells, lymphocytes, and platelets in peripheral blood can recognize the foreign antigens and mount an immune response, which are key hematological parameters of the immune system and reflect the body’s immune function [41]. Cyclophosphamide can cause the acute suppression of hematopoiesis, together with leucopenia, thrombocytopenia, and anemia [42,43]. The influences of UCP on white blood cells, lymphocytes, and platelets in peripheral blood of immunosuppressive mice were investigated. As shown in Figure 5, compared with the normal group, the numbers of white blood cells, lymphocytes, and platelets were significantly decreased in the model group, indicating that the hematopoietic inhibition model was effectively established.

Compared with the model group, after treatment with UCP, the numbers of white blood cells, lymphocytes, and platelets in the blood of the immunosuppressive mice were markedly increased in a dose-dependent manner. At the concentrations of 30 and 60 mg/kg, the effects of UCP on white blood cells and lymphocytes were obvious. In addition, the enhancing of platelets was statistically significant after treatment with UCP (60 mg/kg). It was noted that at the dose of 60 mg/kg, the effects of UCP on white blood cells, lymphocytes, and platelets were similar to those of LH. The results showed that UCP could effectively improve the hematopoietic inhibition caused by cyclophosphamide.

#### 2.5.3. Influence of UCP on Serum Antibody Level

**Figure 6:**
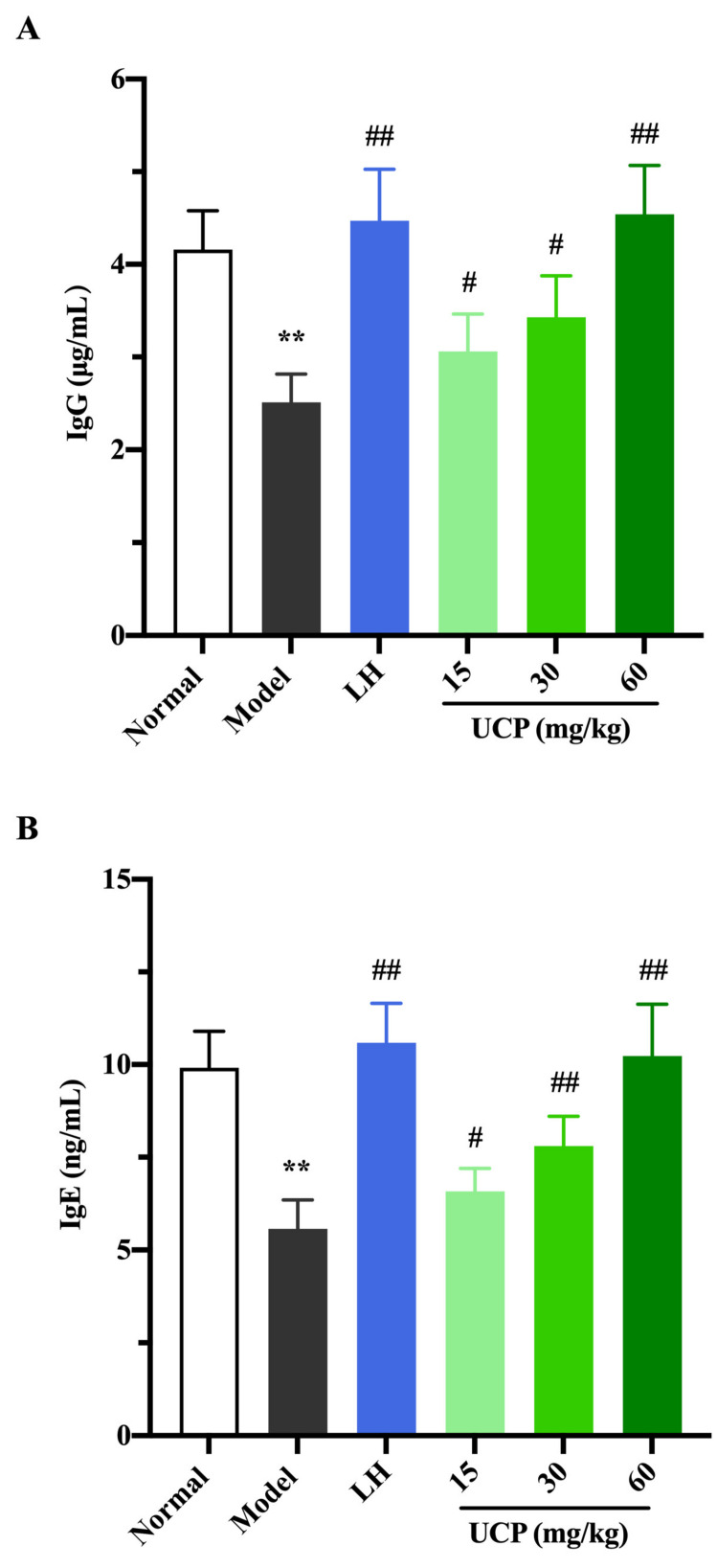

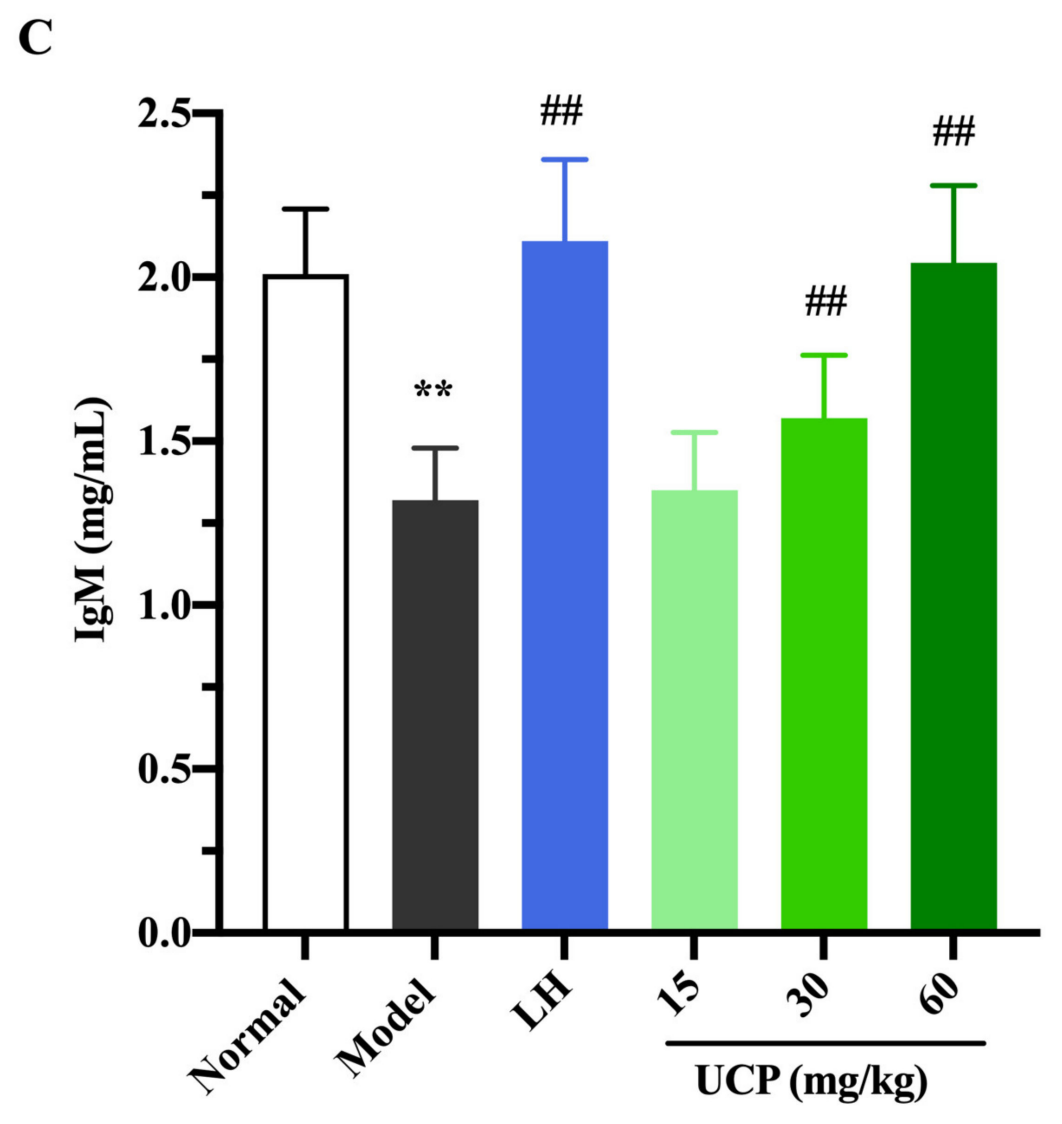
The effects of UCP on the IgG (**A**), IgE (**B**), and IgM (**C**) levels in immunosuppressive mice induced by cyclophosphamide. The data were represented as the means ± SD (*n* = 10). Significant: ** *p* < 0.01 vs. normal group; ^#^ *p* < 0.05, ^##^ *p* < 0.01 vs. model group.

Immunoglobulin (Ig) is a polyclonal antibody and has a direct correlation with the complement activation and neutralization of bacteria or viruses and toxins in the humoral immune response [44,45]. In order to study the effect of UCP on serum antibody levels in immunosuppressive mice, the levels of IgG, IgM, and IgE in serum were determined. As shown in Figure 6, the levels of IgE, IgG, and IgM in serum were significantly decreased when the mice were treated with cyclophosphamide compared with the normal group. UCP noticeably increased the levels of IgG, IgE, and IgM in the serum of the immunosuppressive mice in a dose-dependent manner. At the dose of 60 mg/kg, the levels of IgG, IgE, and IgM in UCP groups were significantly enhanced compared with model groups. Moreover, it was noted that the results were similar to those of LH groups. The data demonstrated that UCP could effectively improve humoral immunity.

Overall, these results indicated that UCP possessed a remarkable immunomodulatory effect in vivo and in vitro. UCP significantly stimulated the proliferation of spleen lymphocytes and increased macrophage phagocytosis in vitro. Furthermore, UCP also significantly increased the thymus and spleen indices and reduced the damage of thymus and spleen tissues caused by cyclophosphamide in vivo. UCP had beneficial effects on hematopoietic function recovery by improving the numbers of white blood cells, lymphocytes, and platelets. UCP could also stimulate immune processes by increasing the levels of antibodies in the immunosuppressive mice. The immune system plays a main role in protecting organisms from infectious disease and metastases through layered defenses with increasing specificity [46]. It is necessary to develop effective functional compounds to modulate the immune system. The spleen and thymus organs are vital immune organs that reflect the immune functions of the host. White blood cells, lymphocyte cells, and platelets in peripheral blood are key hematological parameters of the immune system. Immunoglobulins, such as IgE, IgM, and IgG, as intercellular signaling proteins, are responsible for the immune response, differentiation, and regulation. Our results illustrated that UCP had the potential to develop into a novel immunomodulatory agent for immunity enhancement. It was observed that the increasing effect of UCP on the proliferation of spleen lymphocytes in vitro was stronger than that of the polysaccharide UP0 from *U. lactuca* [47]. Moreover, the increasing effects of UCP on spleen and thymus indices in vivo were higher than those of polysaccharide UPP from *U*. *pertusa* [48]. The structural characteristics of UCP were different from those of UP0 and UPP. Further work is required to investigate the relationship between the fine structure and immunomodulating activity of ulvan with different structures.

Algal polysaccharides possess diverse bioactivities and peculiar chemical structures and represent a great potential source to be explored. Over the last few decades, the functional food and pharmaceutical industries have shown a strong interest in polysaccharides from algae [49]. Polysaccharides from algae have potential as promising candidates for marine drug development. The present results revealed that the sulfated polysaccharide UCP from the green alga *U. conglobata* Kjellman possessed strong immunoregulatory activity in vivo and in vitro. UCP may be a promising immunoregulatory polysaccharide and has potential as a drug or a food supplement for health promotion and immunosuppressive treatment. An in-depth investigation about the immunomodulatory mechanism of activity of UCP is in progress.

## 3. Materials and Methods

### 3.1. Materials

*U. conglobata* Kjellman was collected from the coast of Yantai, China, on May 2016. The raw material was thoroughly washed with tap water, air-dried, milled using a blender, and then stored at room temperature in a dry environment. Q Sepharose Fast Flow and Sephacryl S-400/HR column were from GE Healthcare Life Sciences (Piscataway, NJ, USA). Dialysis membranes (flat width 44 mm, molecular weight cut-off 3500; flat width 31 mm, molecular weight cut-off 1000) were from Lvniao (Yantai, China). Pullulan standards (Mw: 9.6, 21.1, 47.1, 107, 200, 344, and 708 kDa) were from Showa Denko K.K. (Tokyo, Japan). Penicillin, streptomycin, ConA, LPS, l-rhamnose, d-rhamnose, d-xylose, l-xylose, d-glucuronic acid, and l-glucuronic acid were from Sigma (St. Louis, MO, USA). LH was from Aladdin (Shanghai, China). Enzyme linked immunosorbent assay (ELISA) kits for IgG, IgM, and IgE were from Beyotime (Shanghai, China). Dulbecco’s modified Eagle’s medium (DMEM), fetal bovine serum (FBS), and Roswell Park Memorial Institute (RPMI) 1640 were from Lonza (Walkersville, MD, USA).

### 3.2. Cell Culture

RAW264.7 cells obtained from American Type Culture Collection (Manassas, VA, USA, CVCL_0493) were cultured in the following conditions: medium: 10% FBS + DMEM medium + 1% double antibiotics (penicillin and streptomycin); culture environment: 37 °C incubator; saturated humidity: 5% CO_2_.

### 3.3. Animals

BALB/c mice (18–20 g) were housed at 23 ± 2 °C under a 12 h light/dark cycle with free access to food and water. All animal experiments were approved by the Institutional Animal Care and Use Committee of Ocean University of China (OUC-YY-201801001).

### 3.4. Isolation and Purification of the Sulfated Polysaccharide

Alga powder (120 g) was dipped into 50 vols of distilled water and extracted at 100 °C for 4 h and then centrifuged at 3600× *g* for 15 min, concentrated, and dialyzed in a cellulose membrane against distilled water three times. The retained fraction was recovered, concentrated by rotary evaporation, precipitated by adding four volumes of 95% ethanol (*v*/*v*), and then dried at 40 °C to obtain a crude polysaccharide. The crude polysaccharide was fractionated on a Q Sepharose Fast Flow column (30 cm × 3.5 cm) and eluted with a stepwise gradient of 0, 0.5, 1.0, 1.5, 2.0, 2.5, 3.0, and 3.5 mol/L NaCl at a flow rate of 1 mL/min. Elutions were collected by an auto-fraction collector (8 mL/tube, Bio-Rad, Hercules, CA, USA). Total sugar content of the elution was determined by the phenol–sulfuric acid method. The subfraction eluted with 0.5 mol/L NaCl was concentrated and then purified through a Sephacryl S-400/HR column (100 cm × 2.5 cm) eluted with 0.2 mol/L NH_4_HCO_3_ at a flow rate of 0.3 mL/min. Major fractions were collected, concentrated, and freeze-dried (1.74 g).

### 3.5. Structural Characterization of Polysaccharide

The homogeneity and molecular weight were determined by HPGPC on a Shodex OHpak SB-804 HQ column (7.8 mm × 300 mm, Tokyo, Japan) and eluted with 0.2 mol/L Na_2_SO_4_ at a flow rate of 0.5 mL/min. The molecular weight was estimated by reference to a calibration curve made by pullulan standards. Total sugar content was assayed by the phenol–sulfuric acid method using rhamnose as the standard [50]. Protein content was determined as described by a bicinchoninic acid protein assay kit (Shanghai, China). Sulfate ester content was measured according to the method of Therho and Hartiala [51]. Uronic acid content was determined by the carbazole–sulfuric acid method with some modification using glucuronic acid as standard [52]. Monosaccharide compositions were measured by reversed phase HPLC after precolumn derivatization [53]. The calculation of the molar ratio of the monosaccharide was carried out on the basis of the ratio of peak areas of the monosaccharide and the correspondent monosaccharide standard. The sugar configuration was determined according to the method of Tanaka et al. [54]. Identification of the sugar configuration was completed by comparison with retention time of the derivatives of the reference sugars. Desulfation of UCP was performed by reference to Falshaw and Furneaux [55], and the product was named DsUCP. Carboxyl reduction of UCP was carried out according to the method of Taylor and Conrad [56], and the reduction product was designated as RdUCP. Methylation analysis was performed according to the method of Sims et al. [57] with some modification. The methylation products were analyzed by gas chromatography–mass spectrometry (GC–MS) on a TRACE 1300 instrument (Thermo Fisher, Waltham, MA, USA). Identification of partially methylated alditol acetates was carried out on the basis of retention time and mass fragmentation patterns.

### 3.6. Spectroscopy Analysis

The FTIR spectrum of the polysaccharide was measured on a Nicolet Nexus 470 spectrometer (Thermo Fisher Scientific, Waltham, MA, USA) according to the method of Shingel [58]. The polysaccharide was mixed with KBr powder, ground, and pressed into a 1 mm pellet for FTIR measurements in the frequency range of 4000–400 cm^−^^1^. NMR spectra, including ^1^H and ^13^C NMR, ^1^H–^1^H COSY, ^1^H–^13^C HSQC, and ^1^H–^1^H NOESY, were performed at 23 °C through an Agilent DD2 500M NMR spectrometer (Agilent Technologies, Santa Clara, CA, USA). Briefly, polysaccharide (50 mg) was dissolved in deuterium and lyophilized, the procedure was repeated three times, and then it was dissolved in 0.5 mL of 99.97% D_2_O (120 mg/mL). Chemical shifts were expressed in ppm using acetone as an internal standard at 2.225 ppm for ^1^H and 31.07 ppm for ^13^C.

### 3.7. Preparation of Spleen Lymphocytes

The preparation of spleen lymphocytes was carried out according to Yi et al. [59] with some modifications. The spleens of mice were obtained under sterile conditions and chopped into small pieces. Then, the pieces were put into a sterile grinding dish with 1 mL of serum-free RPMI 1640 medium. After gently grinding, the splenocytes were obtained by gently pressing the organ fragments through a 70 μm cell strainer (Solarbio, Beijing, China). The homogeneous cell suspension was resuspended in lysis buffer (0.15 mol/L NH_4_Cl, pH 7.4) for 5 min to remove erythrocytes. Then spleen lymphocytes were harvested after centrifuging at 180× *g* for 10 min and resuspended in RPMI 1640 medium with 10% (*v*/*v*) FBS and 1% antibiotics. Cell numbers and viability (over 95%) were assessed microscopically using the trypan blue dye exclusion technique.

### 3.8. Assay of Spleen Lymphocyte Proliferation

The assay was carried out according to Yu et al. [60]. The spleen lymphocytes were cultured in 96 well plates with a density of 3 × 10^5^ cells/well with or without ConA (5 μg/mL) or LPS (20 μg/mL). After incubation at 37 °C in a humidified atmosphere containing 5% CO_2_ for 48 h, 15 μL of 3-(4,5-dimethylthiazolyl-2)-2,5-diphenyl tetrazoliumbromide (MTT) (5 mg/mL) was added to each well. Then, the plates were incubated at 37 °C for 4 h, and 150 μL of dimethyl sulphoxide (DMSO) was added to resolve the formazan. The optical density (OD) at 570 nm after 15 min was measured using a microplate reader (EL-800; BioTek Instruments, Winooski, VT, USA).

### 3.9. Phagocytic Activity of RAW264.7 Cells

A neutral red assay was implemented to evaluate the phagocytic activity of RAW264.7 [61]. RAW264.7 cells (2 × 10^4^ cells/well) were transferred into 96 well plates and mixed with different concentrations of polysaccharide, and LPS (0.02 μg/mL) was set as blank control. The cells were incubated at 37 °C containing 5% CO_2_ for 24 h. After incubation, the medium was removed, and phosphate buffer saline (PBS) solution was used to wash the remaining medium. Then, 100 μL of neutral red solution (0.08%, *v*/*v*) was added to each well and incubated at 37 °C for 30 min. Then, the supernatant was removed, and each well was washed by PBS solution three times. After that, 150 μL of cell lysis buffer (ethanol:acetic acid = 1:1, *v*/*v*) was added to each well and incubated at 4 °C for 30 min. The OD of each well at 550 nm was recorded, and the well only with lysis buffer was set as the control. The cell phagocytic activity was calculated according to the following equation:

Phagocytic activity = (OD_sample_ − OD_DMSO_)/(OD_blank_ − OD_DMSO_) × 100%

### 3.10. Animal Experimental Design

BALB/c mice (18–20 g) were housed at 23 ± 2 °C under a 12 h light/dark cycle with free access to food and water. After 3 days adaption, BALB/c mice were divided randomly into 6 groups (10 mice in each group) with similar body weight as follows: normal group, model group, LH group (25 mg/kg), and UCP groups (15, 30, or 60 mg/kg). During the period of 5 days, one group was treated with saline as normal control, and the other five groups were treated with cyclophosphamide (45 mg/kg) by intraperitoneal injection (i.p.) once a day to establish the immunosuppressive mouse model. Then, the following 14 days were the experimental stage. The mice of LH and UCP groups were treated with LH (25 mg/kg, i.p.) and UCP (15, 30, or 60 mg/kg, i.p.), respectively, once a day, while the mice of the model and normal control groups were administrated with equivalent amounts of normal saline (i.p.). On the 14th day, the body weight of the mice in the six groups was measured, and then all mice were sacrificed. The blood and tissues needed for further experiments were carefully collected.

### 3.11. Analysis of Spleen and Thymus Indices

The spleen and thymus organs were surgically separated and weighed. The visceral index was calculated according to the following equation: Index (mg/g) = weight of spleen or thymus (mg)/body weight (g).

### 3.12. Peripheral Blood Cell Counts

The whole blood was collected into a blood routine tube by enucleating the eyeball. The contents of white blood cells, lymphocytes, and platelets were analyzed using an automatic hematology analyzer (SYSMEX XN-2800, Sysmex Corporation, Shanghai, China).

### 3.13. Analysis of Immunoglobulins in Serum

The eyeball blood was collected into a fresh sterile tube and kept at 4 °C for 2 h. The tube was centrifuged at 4 °C, 1500× *g* for 10 min to obtain the serum. Then the levels of IgG, IgE, and IgM were detected with ELISA kits according to the instructions of the manufacturer.

### 3.14. Statistical Analysis

Data were expressed as means ± standard deviations (SDs). The statistical significance of difference analysis was performed by using Student’s *t*-test. *p*-values less than 0.05 were considered statistically significant. Statistical significance was denoted by asterisks and hashes, and * and # represent *p* < 0.05, while ** and ## represent *p* < 0.01.

## 4. Conclusions

The sulfated polysaccharide UCP from *U. conglobata* Kjellman was constituted by →4)-α/β-l-Rha*p*-(1→, →4)-β-d-Xyl*p*-(1→, and →4)-β-d-GlcA*p*-(1→ residues, and sulfate groups were at C-3 of→4)-α/β-l-Rha*p*-(1→ and C-2 of →4)-β-d-Xyl*p*-(1→. Moreover, partial glycosylation was at C-2 of →4)-α-l-Rha*p*-(1→ units. The branches contained →4)-β-l-Rha*p*(3SO_4_)-(1→ units, and →4)-β-d-GlcA*p*-(1→ and/or →4)-β-d-Xyl*p*-(1→ units may be also present in the side chains. UCP had potent immunomodulatory activity both in vitro and in vivo. UCP could stimulate lymphocyte proliferation, activate macrophages, and improve the phagocytotic ability. Additionally, UCP increased the levels of peripheral blood cells and serum antibodies, and it also promoted the growth of immune organs and maintained the stability of the internal environment. Therefore, UCP has promise for the development into a drug or a food supplement for health promotion and immunosuppressive treatment. Further study on the action mechanism of UCP deserves to be performed.

## Figures and Tables

**Table 1:** Results of methylation analyses of UCP, DsUCP, and RdUCP.

Methylated Alditol Acetate	Molar Percent Ratio			Linkage Pattern
	UCP	DsUCP	RdUCP	
1,5-Di-*O*-acetyl-2,3,4-tri-*O*-methyl-rhamnitol	2.46	2.56	1.94	Rha*p*-(1→
1,5-Di-*O*-acetyl-2,3,4-tri-*O*-methyl-xylitol	1.69	1.98	1.51	Xyl*p*-(1→
1,5-Di-*O*-acetyl-2,3,4,6-tetro-*O*-methyl-glucitol	--	--	2.43	Glc*p*-(1→
1,4,5-Tri-*O*-acetyl-2,3-di-*O*-methyl-rhamnitol	20.31	59.68	15.37	→4)-Rha*p*-(1→
1,4,5-Tri-*O*-acetyl-2,3-di-*O*-methyl-xylitol	11.08	24.83	8.38	→4)-Xyl*p*-(1→
1,2,4,5-Tetra-*O*-acetyl-3-*O*-methyl-xylitol	13.99	--	10.59	→2,4)-Xyl*p*-(1→
1,2,4,5-Tetra-*O*-acetyl-3-*O*-methyl-rhamnitol	10.07	10.94	7.02	→2,4)-Rha*p*-(1→
1,3,4,5-Tetra-*O*-acetyl-2-*O*-methyl-rhamnitol	40.38	--	30.56	→3,4)-Rha*p*-(1→
1,4,5-Tri-*O*-acetyl-2,3,6-tri-*O*-metyl-glucitol	--	--	22.17	→4)-Glc*p*-(1→

**Table 2:** ^1^H and ^13^C chemical shifts of UCP.

Residues		Chemical Shifts (ppm)					
		1	2	3	4	5	6
A →2,4)-α-l-Rha*p*-(1→	^1^H	5.31	4.44	4.19	3.83	4.17	1.35
	^13^C	102.59	79.80	70.41	79.59	69.61	18.69
B →4)-α-l-Rha*p*(3SO_4_)-(1→	^1^H	5.12	4.26	4.67	3.84	4.05	1.35
	^13^C	103.33	70.52	79.80	79.49	69.61	18.69
C →4)-β-d-Xyl*p*-(2SO_4_)-(1→	^1^H	5.02	4.16	3.90	3.70	3.52	
	^13^C	101.41	79.49	72.72	75.34	62.75	
D →4)-β-l-Rha*p*(3SO_4_)-(1→	^1^H	4.95	4.30	4.62	3.80	3.63	1.35
	^13^C	99.77	70.51	79.80	79.49	71.62	18.69
E →4)-β-l-Rha*p*-(1→	^1^H	4.91	4.21	4.00	3.77	3.60	1.35
	^13^C	98.93	70.4	69.59	79.49	71.62	18.69
F →4)-β-d-GlcA*p*-(1→	^1^H	4.68	3.38	3.68	3.64	3.82	
	^13^C	104.67	75.13	75.16	80.29	77.34	176.46
G →4)-β-d-Xyl*p*-(1→	^1^H	4.55	3.36	3.67	3.68	3.38/4.17	
	^13^C	105.18	75.03	75.43	77.56	63.92	

Spectra were performed on an Agilent DD2 500M NMR spectrometer. Chemical shifts were referenced to internal acetone at 2.225 ppm for ^1^H and 31.07 ppm for ^13^C.

## Data Availability

Not applicable.

## Supplementary Materials

The following are available online at https://www.mdpi.com/article/10.3390/md20070447/s1, Figure S1: NMR spectra of UCP.
