# Supplementary material for: Immunomodulatory Activity In Vitro and In Vivo of a Sulfated Polysaccharide with Novel Structure from the Green Alga Ulva conglobata Kjellman
Source: Mar Drugs. 2022 Jul 8;20(7):447. doi: 10.3390/md20070447 (PMC9320874; doi:10.3390/md20070447)
Supplement: Supplementary file 1 [file marinedrugs-20-00447-s001.zip › marinedrugs-1797554-supplementary.pdf]

SUPPLEMENTARY FIGURES

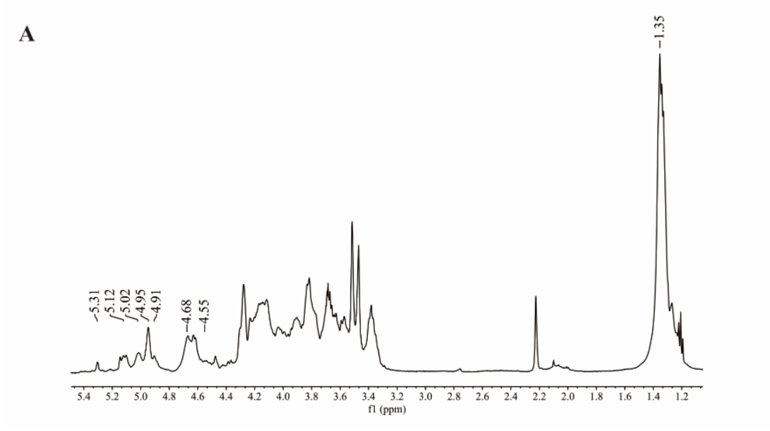

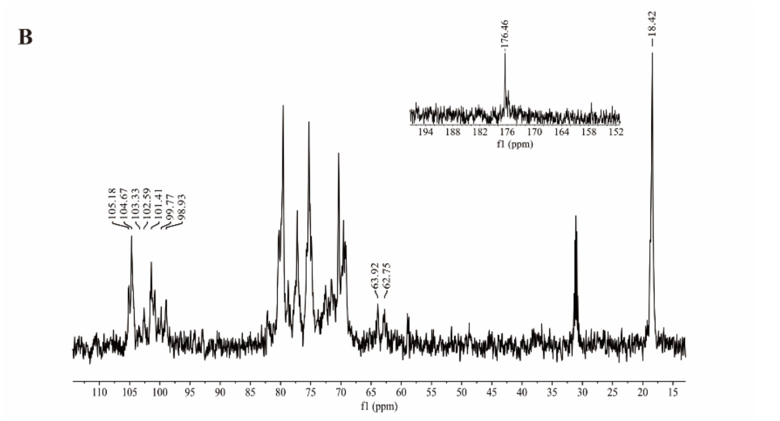

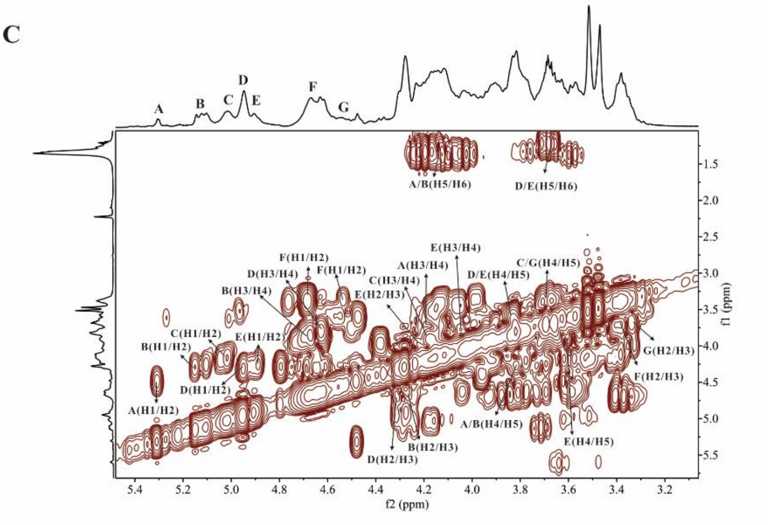

D

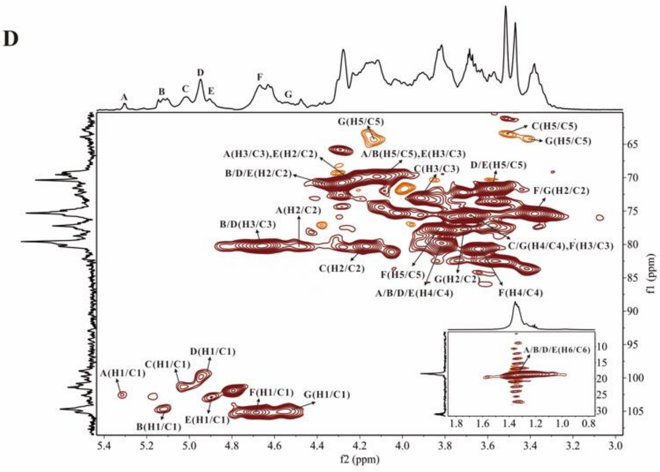

E

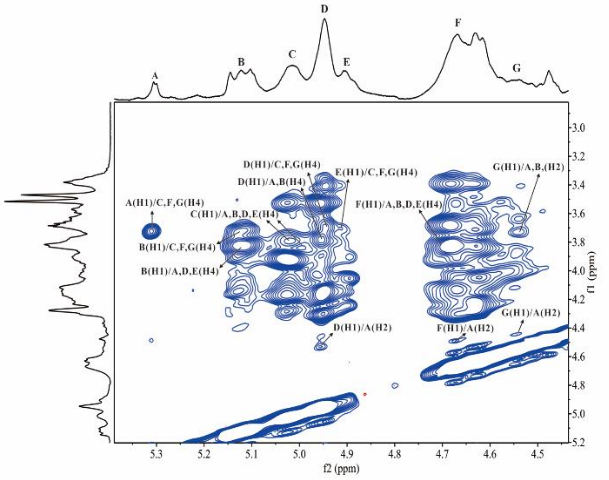

Figure S1. NMR spectra of UCP. Spectra were performed on an Agilent DD2 500M NMR spectrometer. Chemical shifts are referenced to internal acetone at 2.225 ppm for ^{1}H and 31.07 ppm for ^{13}C. (A) ^{1}H NMR spectrum; (B) ^{13}C NMR spectrum; (C) ^{1}H–^{1}H COSY spectrum; (D) ^{1}H–^{13}C HSQC spectrum; and (E) ^{1}H–^{1}H NOESY spectrum. A–G correspond to →2,4)-*α*-L-Rha*p*-(1→, →4)-*α*-L-Rha*p*(3SO_4)-(1→, →4)-β-D-Xyl*p*-(2SO_4)-(1→, →4)-β-L-Rha*p*(3SO_4)-(1→, →4)-β-L-Rha*p*-(1→, →4)-β-D-GlcA*p*(1→ and →4)-β-D-Xyl*p*-(1→, respectively.
